# Epigenetics of type 2 diabetes and diabetes-related outcomes in the Strong Heart Study

**DOI:** 10.1186/s13148-022-01392-7

**Published:** 2022-12-18

**Authors:** Arce Domingo-Relloso, Matthew O. Gribble, Angela L. Riffo-Campos, Karin Haack, Shelley A. Cole, Maria Tellez-Plaza, Jason G. Umans, Amanda M. Fretts, Ying Zhang, M. Daniele Fallin, Ana Navas-Acien, Todd M. Everson

**Affiliations:** 1 Department of Chronic Diseases Epidemiology, National Center for Epidemiology, Carlos III Health Institute, Madrid, Spain; 2 Department of Environmental Health Sciences, Columbia University Mailman School of Public Health, New York, NY USA; 3 Department of Statistics and Operations Research, University of Valencia, Valencia, Spain; 4 Department of Epidemiology, University of Alabama at Birmingham School of Public Health, Birmingham, AL USA; 5 Millennium Nucleus On Sociomedicine (SocioMed) and Vicerrectoría Académica, Universidad de La Frontera, Temuco, Chile; 6 Department of Computer Science, ETSE, University of Valencia, Valencia, Spain; 7 Population Health Program, Texas Biomedical Research Institute, San Antonio, TX USA; 8 MedStar Health Research Institute, Hyattsville, MD USA; 9 Georgetown-Howard Universities Center for Clinical and Translational Science, Washington, DC USA; 10 Department of Epidemiology, Cardiovascular Health Research Unit, University of Washington, Seattle, WA USA; 11 Department of Biostatistics and Epidemiology, The University of Oklahoma Health Sciences Center, Oklahoma City, OK USA; 12 Emory University Rollins School of Public Health, Atlanta, GA USA; 13 Gangarosa Department of Environmental Health, Emory University Rollins School of Public Health, Atlanta, GA USA; 14 Department of Epidemiology, Emory University Rollins School of Public Health, Atlanta, GA USA

**Keywords:** Epigenetics, Type 2 diabetes, DNA methylation, American Indians

## Abstract

**Background:**

The prevalence of type 2 diabetes has dramatically increased in the past years. Increasing evidence supports that blood DNA methylation, the best studied epigenetic mark, is related to diabetes risk. Few prospective studies, however, are available. We studied the association of blood DNA methylation with diabetes in the Strong Heart Study. We used limma, Iterative Sure Independence Screening and Cox regression to study the association of blood DNA methylation with fasting glucose, HOMA-IR and incident type 2 diabetes among 1312 American Indians from the Strong Heart Study. DNA methylation was measured using Illumina’s MethylationEPIC beadchip. We also assessed the biological relevance of our findings using bioinformatics analyses.

**Results:**

Among the 358 differentially methylated positions (DMPs) that were cross-sectionally associated either with fasting glucose or HOMA-IR, 49 were prospectively associated with incident type 2 diabetes, although no DMPs remained significant after multiple comparisons correction. Multiple of the top DMPs were annotated to genes with relevant functions for diabetes including *SREBF1*, associated with obesity, type 2 diabetes and insulin sensitivity; *ABCG1*, involved in cholesterol and phospholipids transport; and *HDAC1*, of the HDAC family. (HDAC inhibitors have been proposed as an emerging treatment for diabetes and its complications.)

**Conclusions:**

Our results suggest that differences in peripheral blood DNA methylation are related to cross-sectional markers of glucose metabolism and insulin activity. While some of these DMPs were modestly associated with prospective incident type 2 diabetes, they did not survive multiple testing. Common DMPs with diabetes epigenome-wide association studies from other populations suggest a partially common epigenomic signature of glucose and insulin activity.

**Supplementary Information:**

The online version contains supplementary material available at 10.1186/s13148-022-01392-7.

## Introduction

The burden of diabetes has dramatically increased worldwide over the past decades, leading to excessive health, social and economic costs [1, 2]. Diabetes is one of the main risk factors for cardiovascular disease [3] and other complications such as kidney disease, blindness, cancer and liver disease [1]. Most of the Genome-Wide Association Studies (GWAS) of diabetes have been conducted in European populations [4]. The loci identified as associated with T2D were validated in other ethnicities such as Mexican Americans [5]. However, the identified loci only explain about 20% of the heritability of T2D in Europeans [6]. Environmental factors, including diet, physical activity, obesogenic chemicals and their impact in excess adiposity, are major determinants of diabetes [7]. These environmental factors, together with genetics, are in turn major regulators of epigenetic marks [8], supporting that epigenetics provides a framework to advance our understanding of relevant pathways for diabetes and diabetes-related outcomes.

DNA methylation refers to the covalent attachment of a methyl group to the DNA molecule [9]. Several studies have highlighted the association between blood DNA methylation and diabetes [10–13]; however, the temporality between DNA methylation dysregulations and diabetes is unclear and those dysregulations might also be a consequence of metabolic processes induced by subclinical diabetes. In addition, DNA methylation dysregulations associated with diabetes have also been found in other relevant tissues such as liver, adipose tissue and pancreatic islets [14]. As the prediabetes period is long and the diabetes diagnostic criteria are established in advanced phases, DNA methylation dysregulations might be a powerful biomarker for early detection [15], which might help with early treatment and to reduce healthcare costs. In addition, DNA methylation has been suggested to have a higher predictive ability than genetics for type 2 diabetes in high-risk subjects [16].

Native Americans suffer a disproportionate burden of diabetes compared to other race/ethnic groups in the USA [17]. American Indian and Alaska Native adults are almost three times more likely than non-Hispanic white adults to be diagnosed with diabetes [18]. The Strong Heart Study [19] is the largest prospective cohort study of cardiovascular disease and its risk factors in American Indian communities and provides an opportunity to evaluate the association of DNA methylation with diabetes in a population with a high burden of metabolic disease.

Most of the previous epigenome-wide association studies (EWAS) of diabetes have been cross-sectional, have evaluated associations of DNA methylation with prevalence rather than incidence, have been conducted among individuals with clinically diagnosed diabetes or lack a time-to-event framework [10–13, 20]. Comparing epigenetic signatures associated cross-sectionally with subclinical markers of diabetes and those associated prospectively with diabetes incidence can contribute to better understand the natural history of diabetes development. We first examined the cross-sectional association between markers of glucose and insulin sensitivity (fasting glucose and HOMA-IR) and blood DNA methylation measured at almost 800,000 genomic loci. Second, we determined whether those epigenetic variations were also associated with incident diabetes in participants of the Strong Heart Study that were free of diabetes at baseline.

## Methods

### Study population

The SHS is the largest and longest prospective cohort study of American Indians. It was funded to study cardiovascular diseases and risk factors in American Indian adults [19]. In 1989–1991 (visit 1), 4549 men and women between 45 and 75 years old belonging to 13 tribes from Arizona, Oklahoma and North Dakota and South Dakota agreed to participate. In 2016, a Tribal Nation from Arizona declined further participation, leaving 3517 potential participants for this study. Eligibility for blood DNA methylation analysis has been described in a previous publication [21], leaving 2325 participants for epigenetic research. For this study, 967 participants with prevalent diabetes (assessed by whether participants took diabetes medication, by measurements of fasting glucose and by the HbA1c test) and 46 participants with missing diabetes information at exam 2 (1993–1995) and exam 3 (1998–1999) or missing values in fasting glucose or HOMA-IR at baseline were excluded, leaving a total of 1312 participants (Additional file 1: Figure S1). The Strong Heart Study protocol was approved by institutional review boards, participating tribal communities and the respective area Indian Health Service IRBs in each tribal community, and all participants provided informed consent.

### Participant characteristics

Data on sociodemographic factors, medical history, smoking status and alcohol consumption were collected in a personal interview. Participants who reported smoking < 100 cigarettes in their lifetime were considered never smokers. Participants who reported smoking ≥ 100 cigarettes in their lifetime and smoking at the time of the interview were considered current smokers. Participants who reported smoking ≥ 100 cigarettes in their lifetime but currently not smoking were classified as former smokers. Current alcohol consumption was defined as self-report of any alcohol consumption within the past year. Former alcohol consumption was defined as no consumption of any alcohol during the last year but previous consumption of > 12 drinks of alcohol in a single year. The physical examination included anthropometric measures (height and weight) and collected fasting blood and spot urine samples.

### Fasting glucose, insulin resistance and incident diabetes

Participants fasted for 12 h or more before the physical examination. An oral glucose tolerance test was conducted, which included 75-g oral glucose and blood collection at 2 h [22]. The MedStar Health Research Institute laboratory [22, 23] analyzed plasma glucose and insulin using a hexokinase method and radioimmunoassay (Linco, St. Louis, Missouri), respectively. The analysis of hemoglobin A1c (HbA1c) in blood was conducted at the National Institute of Diabetes and Digestive and Kidney Diseases Epidemiology and Clinical Research Branch, Phoenix, Arizona using high-performance liquid chromatography [24].

We defined diabetes as having one of the following: fasting glucose greater or equal than 126 mg/dL, 2-h post-load plasma glucose greater or equal than 200 mg/dL, HbA1c greater or equal than 6.5%, using insulin or an oral hypoglycemic agent [25]. We used the equation [fasting plasma insulin (mU/L) × fasting plasma glucose (mmol/L)]/22.5 to calculate HOMA-IR [26]. Individuals having prevalent diabetes were excluded, and incident diabetes was assessed in two follow-up visits (1993–1995 and 1998–1999) (Additional file 1: Figure S1).

### Blood DNA methylation determinations

Details of the DNA methylation analysis from white blood cells using Illumina’s MethylationEPIC BeadChip (850 K) have been published [27]. Buffy coats from fasting blood samples were collected in 1989–1991. Biological specimens were stored at − 70 °C. DNA from white blood cells was extracted and stored at the Penn Medical Laboratory, MedStar Health Research Institute under a strict quality-control system. In 2015, blood DNA was shipped to the analytical laboratory at the Texas Biomedical Research Institute for DNAm analysis. DNA was bisulfite-converted with the EZ DNAm kit (Zymo Research) according to the manufacturer’s instructions. Bisulfite-converted DNA was measured using the Illumina MethylationEPIC BeadChip (850 K). CpGs with a p-detection value greater than 0.01 in more than 5% of the individuals (6159 CpGs) were removed. Single sample normalization for background correction [28] and Regression on Correlated Probes normalization for probe type bias [29] were applied. We excluded cross-hybridizing DNA methylation sites, sex chromosomes DNA methylation sites and single nucleotide polymorphisms with minor allele frequency > 0.05 [30]. We calculated blood cell proportions (CD8T, CD4T, NK cells, B cells, monocytes and neutrophils) using the Houseman method as implemented by the R package FlowSorted.Blood.EPIC. We used those data to adjust all models for blood cell type proportions. The final sample size after these corrections was of 1312 participants. The total number of CpG sites available was 788,368 CpG sites.

### Statistical analysis

#### Cross-sectional association: one marker at a time

We conducted an epigenome-wide association study to evaluate the association of each CpG site separately with both fasting glucose and HOMA-IR (in separate models). We used linear regression models as implemented by the limma R package, together with an empirical Bayes method that shrinks standard errors to a common value in order to borrow information from all the genes. We used methylation M values (logit2 transformation of methylation proportions) as the outcome. Models were adjusted for age, sex, study center, smoking status (never, former, current), alcohol consumption (never, former, current), education level (no high school, some high school, completed high school), BMI, estimated cell proportions (CD8T, CD4T, NK, B cells and monocytes) and five genetic principal components (PCs). *P*-values were corrected for multiple comparisons using the FDR method.

#### Cross-sectional association: all markers at a time

We used Iterative Sure Independence Screening coupled with Adaptive Elastic-Net (ISIS-AENET) to analyze the association of the 788,368 CpGs simultaneously with fasting glucose or HOMA-IR. ISIS-AENET uses data-driven weights to improve effect estimation while allowing to analyze highly correlated predictors, similar to elastic-net models [31, 32]. The combination of ISIS and regularization methods has shown to be a highly effective variable selection method relative to other approaches in ultra-high-dimensional settings [31, 33, 34]. A combination of the R packages SIS [35] and gcdnet [36] was used to perform this analysis. To remove the variability in methylation due to differences in cell type proportions, methylation data were regressed on cell type proportions (CD8 T cells, CD4 T cells, NK cells, B cells and monocytes) and five genetic principal components (PCs) and the residuals of those models were used as predictors in ISIS-AENET models. In addition, the two outcomes (fasting glucose and HOMA-IR) were separately regressed on relevant baseline covariates (age, sex, study center, smoking status (never, former, current), alcohol consumption (never, former, current), education level (no high school, some high school or completed high school) and BMI, and the residuals of those models were used as outcomes in the ISIS-AENET model.

#### Prospective association with incident diabetes

The beta values (DNA methylation proportions) of the differentially methylated CpG positions (DMPs) selected by the ISIS-AENET model as associated with either fasting glucose or HOMA-IR were evaluated for prospective association with incident diabetes using Cox proportional hazards models of one CpG at a time. We used diabetes status at visits 2 and 3 to determine the time to event, with age as time scale and individual entry times (age at baseline) treated as staggered entries. Models were adjusted for sex, smoking status, study center, alcohol consumption, education level, cell counts and five genetic PCs. We conducted two sensitivity analyses additionally adjusting the models for fasting glucose and for HOMA-IR. As a secondary analysis, we assessed the association between DNA methylation and incident impaired glucose tolerance (IFG) on the CpG sites associated with incident type 2 diabetes. IFG was defined as two-hour glucose levels of 140 to 199 mg/dL on the 75-g oral glucose tolerance test. We used IFG status at visits 2 and 3 to determine the time to event, with age as time scale and individual entry times treated as staggered entries. Models were adjusted for the same covariates as diabetes models.

#### Protein–protein interaction network

We displayed a protein–protein interaction network for the CpGs selected by ISIS-AENET for either fasting glucose or HOMA-IR based on their annotation to the nearest protein coding gene. We used the STRING database v11.0 to identify the interactions between nodes that had a confidence score of at least 0.4 [37]. We used Cytoscape v3.8.2 to display the network [38].

#### Enrichment analysis

We performed functional enrichment analysis on the genes that were annotated to all CpGs selected via the ISIS-AENET models for either fasting glucose or HOMA-IR. We used the *clusterProfiler* R package [39] to test for over-representation of GO terms [40] in the biological pathways (BP), molecular functions (MF) and cellular component (CC) databases, for biological pathways in the Kyoto Encyclopedia of Genes and Genomes (KEGG) [41] and Reactome [42] databases, and for hallmark gene sets from the Molecular Signatures Database (MSigDB) [43]. We present the top 20 enriched pathways and gene sets, ranked by *p*-values from the hypergeometric test, and use false discovery rate (FDR) < 0.05 to distinguish significant enrichment.

## Results

At baseline, SHS participants who developed incident diabetes were younger than those who remained diabetes-free through the follow-up and had higher BMI. They were less likely to be current smokers and drink alcohol and had higher HOMA-IR (Table 1).

**Table 1:** Participants’ characteristics by diabetes status at follow-up

	Incident diabetes (*N* = 348)	Non-diabetes (*N* = 964)
Age (years), median (IQR)	52.9 (48.4, 60.2)	54.3 (48.7, 61.2)
Sex, % Male	39.7	45.5
Smoking status, %		
Former	34.2	28.8
Current	36.8	44.2
BMI, median (IQR)	30.4 (27.3, 34.5)	27.8 (24.8, 31.2)
Alcohol consumption, %		
Former	14.9	11.6
Current	42.8	50.1
Education level, %		
Some high school	22.4	21.6
High school or more	61.8	63.3
Fasting glucose, mg/dL	104 (97, 111)	99 (93, 106)
HOMA-IR	4.0 (2.5, 6.2)	2.5 (1.5, 4.0)

Medians (IQR) or percentages are shown for continuous or categorical variables, respectively

*IQR* interquartile range

The one-marker-at-a-time approach identified one DMP (annotated to the *DNAH10* gene) associated with fasting glucose after multiple comparisons. In models not adjusted by BMI, we found three more DMPs (annotated to *ABCG1, SREBF1* and *DNAH10*). For HOMA-IR, no DMPs were significant at 0.05 FDR threshold. In models not adjusted for BMI, six DMPs (annotated to genes *ABCG1, SREBF1, HSF4* and *CPT1A*) were significant.

The ISIS-AENET model identified 182 DMPs as associated with fasting glucose (Additional file 2: Table S1) and 182 DMPs as associated with HOMA-IR (Additional file 2: Table S2), with an overlap of six DMPs annotated to the genes *MRPS31* (associated with type 1 diabetes in previous studies), *SH2B1* (associated with severe obesity and insulin resistance)*, ABCG1* (associated with type 2 diabetes), *ABHD11* (involved in weight gain regulation), *PSMF1* (control of proteasome function) and *HMGN1* (associated with the process of transcriptionally active chromatin).

Among the 358 DMPs that were associated either with fasting glucose or with HOMA-IR, 49 were associated with incident diabetes at a nominal *p* value of 0.05. None of them passed the FDR cutoff of 0.05. The 16 DMPs associated with incident diabetes at a nominal *p* value < 0.01 are shown in Table 2. Adjustment for baseline fasting glucose or HOMA-IR in these models attenuated the effect estimates. The CpG cg06500161, annotated to the gene *ABCG1*, which was one of the overlapping DMPs between fasting glucose and HOMA-IR, was in the top five DMPs for incident diabetes, showing a strong positive association (Table 2). The other overlapping DMPs for fasting glucose and HOMA-IR did not reach statistical significance for incident diabetes. The two top signals for incident diabetes, annotated to genes *SREBF* and *ABCG1*, were also associated with IFG, with hazard ratios (95% CI-s) 2.4 (1.2, 5.1) and 1.9 (1.1, 3.6), respectively.

**Table 2:** CpGs prospectively associated with incident diabetes in a Cox proportional hazards model at a nominal *p* value < 0.01

CpG	Chr	Gene	Function	Adjusted for covariates^a^		Adjusted for covariates + fasting glucose		Adjusted for covariates + HOMA-IR	
				HR (95% CI)	*p* value	HR (95% CI)	*p* value	HR (95% CI)	*p* value
cg11024682	17	*SREBF1*	Obesity, type 2 diabetes and insulin sensitivity	1.92 (1.35, 2.74)	0.0003	1.66 (1.16, 2.37)	0.005	1.66 (1.17, 2.35)	0.005
cg07750706	6	*PLAGL1*	Transient neonatal diabetes mellitus	0.63 (0.47, 0.83)	0.0009	0.67 (0.51, 0.89)	0.005	0.66 (0.5, 0.87)	0.003
cg23053438	3	*HEG1*	Regulator of heart and vessel formation	0.64 (0.49, 0.83)	0.001	0.66 (0.51, 0.87)	0.003	0.67 (0.51, 0.88)	0.004
cg01885480	15	*OAZ2*	Polyamine biosynthesis, type 2 diabetes	0.64 (0.49, 0.84)	0.001	0.67 (0.51, 0.88)	0.003	0.69 (0.53, 0.9)	0.007
cg06500161	21	*ABCG1*	Cholesterol and phospholipids transport	1.54 (1.16, 2.03)	0.003	1.38 (1.03, 1.84)	0.03	1.27 (0.95, 1.69)	0.11
cg22710955	7	*POP7*	Ribosome biogenesis	0.66 (0.5, 0.87)	0.003	0.70 (0.53, 0.92)	0.01	0.69 (0.52, 0.91)	0.009
cg20463945	6	*C6orf136*	Uncharacterized function	0.70 (0.54, 0.9)	0.006	0.75 (0.58, 0.97)	0.03	0.69 (0.54, 0.9)	0.006
cg15269194	7	*FAM3C*	Type 2 diabetes and non-alcoholic fatty liver disease	0.70 (0.54, 0.9)	0.006	0.75 (0.59, 0.97)	0.03	0.69 (0.53, 0.9)	0.006
cg27089547	16	*ZNF747*	Uncharacterized function	0.69 (0.52, 0.9)	0.006	0.72 (0.55, 0.94)	0.02	0.70 (0.54, 0.92)	0.01
cg20595300	9	*UHRF2*	Cell-cycle regulation, tumorigenesis	0.70 (0.54, 0.91)	0.007	0.75 (0.58, 0.97)	0.03	0.74 (0.57, 0.95)	0.02
cg19048496	16	*TCEB2*	Transcription elongation and cellular senescence	0.69 (0.53, 0.9)	0.007	0.70 (0.53, 0.91)	0.007	0.73 (0.56, 0.96)	0.02
cg08728520	20	*COMMD7*	NF-kappa-B complex activity	0.68 (0.51, 0.9)	0.007	0.70 (0.53, 0.93)	0.01	0.69 (0.51, 0.91)	0.009
cg06935581	16	*DECR2*	Lipid metabolism	0.68 (0.51, 0.9)	0.007	0.70 (0.53, 0.93)	0.01	0.70 (0.52, 0.92)	0.01
cg00291981	3	*BSN*	Spatial organization of synaptic vesicle cluster	0.69 (0.53, 0.91)	0.007	0.72 (0.55, 0.94)	0.02	0.70 (0.54, 0.92)	0.009
cg25944898	11	*PRDX5*	Cellular protection against oxidative stress	0.69 (0.52, 0.91)	0.008	0.71 (0.54, 0.93)	0.01	0.71 (0.54, 0.94)	0.02
cg27555392	1	*FBXO42*	Protein-ubiquitin ligases	0.69 (0.53, 0.91)	0.009	0.72 (0.55, 0.95)	0.02	0.71 (0.54, 0.94)	0.02

Model adjusted for age, smoking status (never, former, current), alcohol consumption (never, former, current), sex, education level (less than high school, some high school, high school or more), Houseman cell proportions (CD8T, CD4T, NK, B cells and monocytes), five genetic PCs and study center (Arizona, Oklahoma or Dakota)

**Fig. 1:**
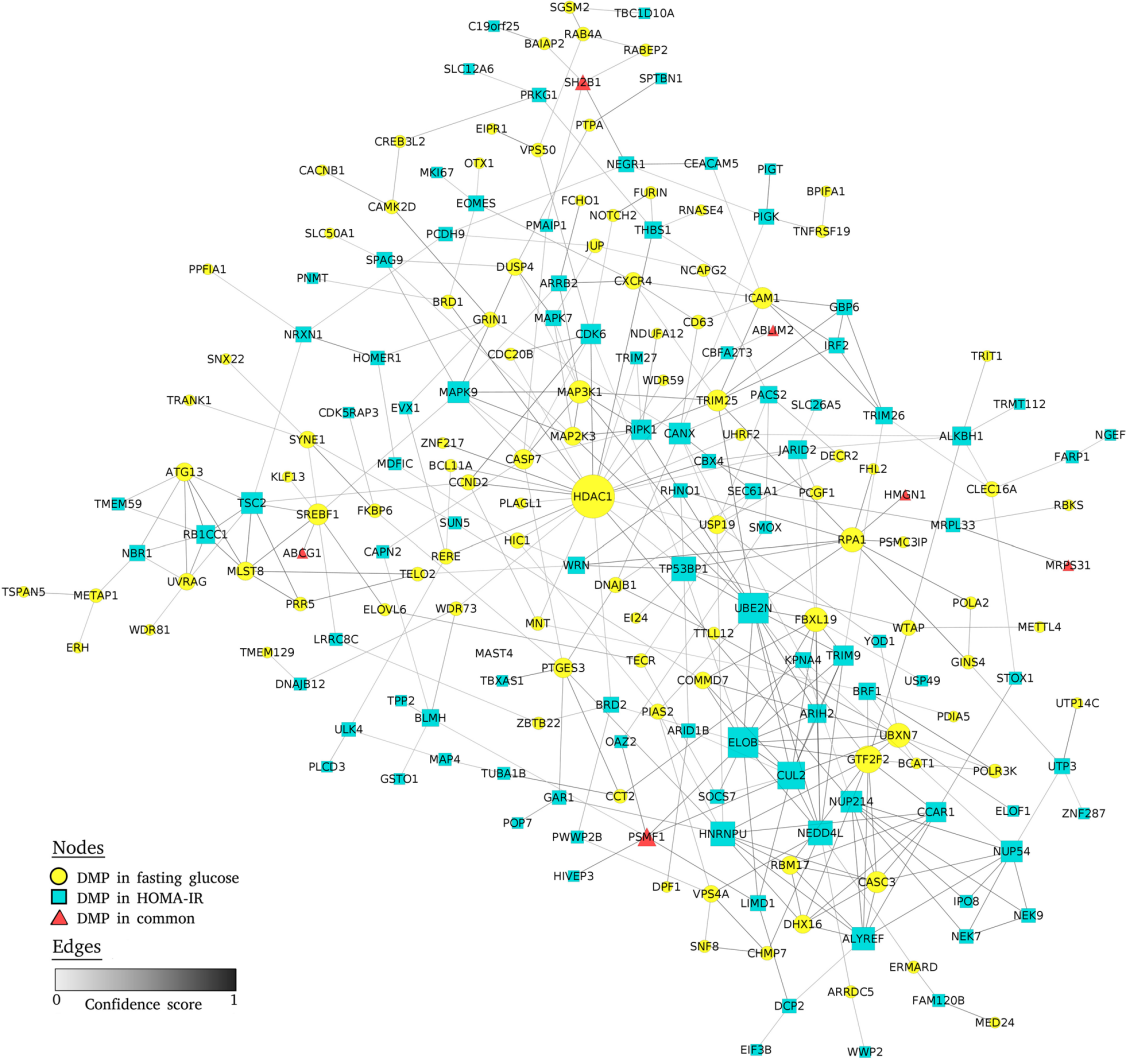
Protein–protein interaction network for fasting glucose and HOMA-IR

In the protein–protein interaction network using the 358 CpGs that were selected by the ISIS-AENET model, a network with 203 nodes and 360 connections was obtained. The hub node was *HDAC1* (differentially methylated for fasting glucose), with 23 interactions, followed by *ELOB* and *UBE2N* nodes (both of them differentially methylated for HOMA-IR), with 14 interactions each (Fig. 1).

**Fig. 2:**
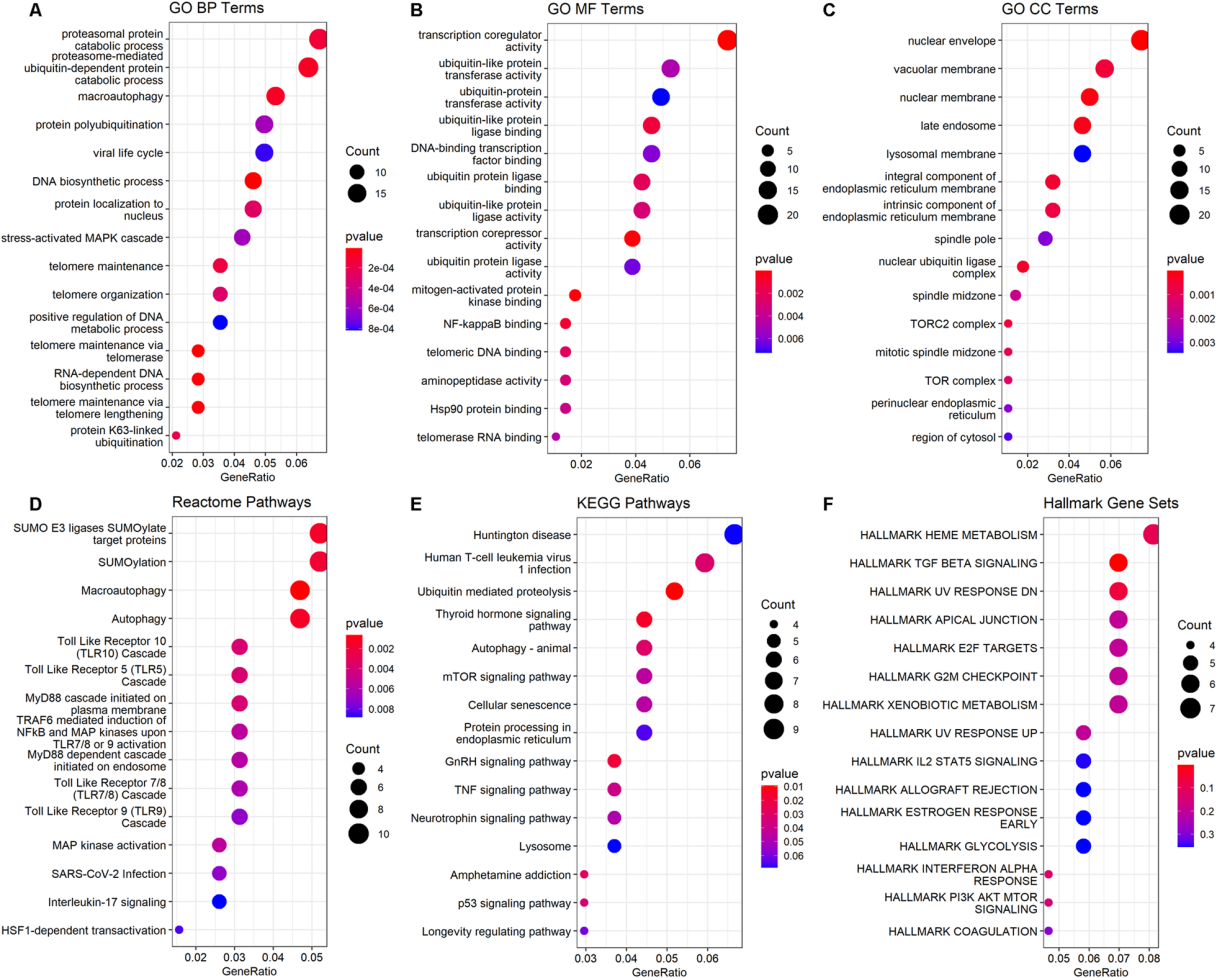
Top 15 GO Terms (**A–C**), pathways (**D–E**) and gene sets (**F**). The x-axis represents the ratio genes from our fasting glucose and HOMA-IR set that were also within the gene set listed on the y-axis; the count represents the number of genes in our fasting glucose and HOMA-IR set that were within each gene set listed on the y-axis, and the color coding represents the *p* values from the hypergeometric tests, with red indicating smaller and blue indicating larger *p*-values

The 358 CpGs that were selected by the ISIS-AENET models for fasting glucose or HOMA-IR were annotated to 303 genes with Entrez Gene identifiers. We highlight the top 15 GO terms (Fig. 2A–C), KEGG and Reactome pathways (Fig. 2D, E) and hallmark gene sets (Fig. 2F) that were over-represented among these 303 genes. The pathways and gene sets that were significantly enriched with FDR < 0.05 (Table 3) tended to represent DNA and RNA biosynthesis and maintenance, telomerase activity, autophagy, transcriptional regulation and upregulated genes in response to transforming growth factor beta 1 (*TGFB1*).

**Table 3:** Significantly over-represented gene sets (FDR < 0.05) among the 303 genes annotated to CpGs associated with fasting glucose and HOMA-IR

Database	ID	Description	*q* val	Gene IDs
GO BP	GO:0071897	DNA biosynthetic process	0.012	*HNRNPU, TELO2, DCP2, PTGES3, NEK7, ARRB2, WRN, RPA1, CCT2, TRIM25, SH2B1, GAR1, PARP10*
GO BP	GO:0007004	Telomere maintenance via telomerase	0.012	*HNRNPU, TELO2, DCP2, PTGES3, NEK7, RPA1, CCT2, GAR1*
GO BP	GO:0006278	RNA-dependent DNA biosynthetic process	0.016	*HNRNPU, TELO2, DCP2, PTGES3, NEK7, RPA1, CCT2, GAR1*
GO BP	GO:0010833	Telomere maintenance via telomere lengthening	0.020	*HNRNPU, TELO2, DCP2, PTGES3, NEK7, RPA1, CCT2, GAR1*
GO BP	GO:0016236	Macroautophagy	0.039	*WDR81, PACS2, MLST8, TSC2, SNF8, RB1CC1, YOD1, FEZ2, NBR1, UVRAG, EI24, ATP6V1H, VPS4A, CLEC16A, ATG13*
GO BP	GO:0043161	Proteasome-mediated ubiquitin-dependent protein catabolic process	0.039	*ANAPC11, MAPK9, ARRB2, CDC20B, PSMF1, TRIM9, FBXL19, CUL2, USP19, TRIM25, ANAPC2, TMEM129, YOD1, DNAJB12, WWP2, ARIH2, CBFA2T3, NEDD4L*
GO MF	GO:0003712	Transcription coregulator activity	0.011	*JUP, HNRNPU, TRIM27, PSMC3IP, HIPK2, TRIM52, LIMD1, FHL2, HDAC1, FBXL19, CCAR1, CBX4, TRIM25, TCF25, RERE, DPF1, TP53BP1, MED24, LPXN, PARP10, CBFA2T3*
GO MF	GO:0051019	Mitogen-activated protein kinase binding	0.024	*MAPK7, ARRB2, DUSP4, CDK5RAP3, NBR1*
GO MF	GO:0003714	Transcription corepressor activity	0.024	*HNRNPU, HIPK2, LIMD1, FHL2, HDAC1, CCAR1, CBX4, TCF25, RERE, PARP10, CBFA2T3*
GO CC	GO:0005635	Nuclear envelope	0.001	*TRIM27, TNPO3, KPNA4, SLC22A18, WTAP, CHMP7, ALG14, CCAR1, P2RX5, CCND2, RB1CC1, EI24, DNAJB12, OSBPL6, NRXN1, NUP214, SYNE1, SREBF1, CASC3, SMOX, POM121L12*
GO CC	GO:0031965	Nuclear membrane	0.019	*TRIM27, KPNA4, WTAP, ALG14, P2RX5, CCND2, RB1CC1, EI24, DNAJB12, OSBPL6, NRXN1, SYNE1, CASC3, SMOX*
GO CC	GO:0005770	Late endosome	0.023	*WDR81, CHMP7, CTNS, SNF8, CD63, LAMP3, NBR1, UVRAG, TMEM59, VPS4A, ARL8B, CLEC16A, NEDD4L*
GO CC	GO:0000152	Nuclear ubiquitin ligase complex	0.030	*ANAPC11, CDC20B, CBX4, ANAPC2, PCGF1*
GO CC	GO:0030176	Integral component of endoplasmic reticulum membrane	0.030	*TECR, FICD, SEC61A1, PIGK, PIGT, CANX, SGMS2, ELOVL6, DNAJB12*
GO CC	GO:0005774	Vacuolar membrane	0.030	*WDR81, VASN, SLC44A2, WDR59, DAGLB, CTNS, SPAG9, CD63, RB1CC1, LAMP3, UVRAG, TMEM59, ATP6V1H, VPS4A, ARL8B, CLEC16A*
GO CC	GO:0031932	TORC2 complex	0.030	*TELO2, MLST8, PRR5*
GO CC	GO:0031227	Intrinsic component of endoplasmic reticulum membrane	0.030	*TECR, FICD, SEC61A1, PIGK, PIGT, CANX, SGMS2, ELOVL6, DNAJB12*
GO CC	GO:1990023	Mitotic spindle midzone	0.039	*HNRNPU, RCC2, NUMA1*
GO CC	GO:0038201	TOR complex	0.043	*TELO2, MLST8, PRR5*
Hallmark	TGF Beta Signaling	TGF Beta Signaling	0.024	*HIPK2, SPTBN1, THBS1, HDAC1, FURIN, FNTA*

## Discussion

In this EWAS of blood DNA methylation, we identified several DMPs prospectively associated with type 2 diabetes in an American Indian population across the Southwest and the Northern Plains of the USA. Using statistical methods that allow the joint evaluation of high-dimensional and highly correlated epigenetic markers, we found 182 DMPs associated with fasting glucose and 182 DMPs associated with HOMA-IR. Of those, 49 were associated with incident diabetes at a nominal *p*-value of 0.05. No DMPs remained significant after correction for multiple comparisons. The bioinformatics analyses pointed to several important regulatory biological pathways for type 2 diabetes, such as autophagy.

Many of the genes annotated to the top DMPs in our EWAS in both the one marker at a time and the multiple markers at a time approaches have biological functions related to type 2 diabetes. The *SREBF1* gene is associated with obesity, type 2 diabetes and insulin sensitivity, and the gene *ABCG1* is involved in cholesterol and phospholipids transport. Importantly, these two genes have been identified in other diabetes EWAS conducted in Indian Asian [44], Mexican American [10] and European [45] populations and have been proposed as potential valuable markers for personalized type 2 diabetes risk prediction [46]. The fact that these two genes were among the top DMPs identified in our study provides evidence in favor of a common epigenomic signature of type 2 diabetes across populations.

However, to our knowledge, this is the first study that investigates the association between blood DNA methylation with type 2 diabetes prospectively. According to the meta-analysis conducted by Raciti et al. [16], which was published by the end of 2021, all the previous studies in blood DNA methylation were either cross-sectional [10, 44, 47–50], focused on targeted genes rather than epigenome-wide [46, 51, 52], or focused on global DNA methylation, rather than site-specific methylation [53]. Although an EWAS conducted in a European population [45] evaluated incident diabetes, they did not account for time to event in the EWAS models, which were conducted using a logistic regression (with a dichotomous outcome) rather than survival analysis.

Several genes annotated to top DMPs in our study also with known diabetes-related functions have not been identified in previous blood EWAS, which suggests that some of the epigenetic markers that are related to diabetes might be population-specific or the high burden of diabetes in our study population enabled the identification of those signals. For example, the gene *PLAGL1* is associated with neonatal diabetes mellitus [54]; *FAM3C* is a therapeutical target for type 2 diabetes and non-alcoholic fatty liver disease [55]; *OAZ2* is differentially methylated in children exposed to maternal diabetes in utero versus unexposed [56]; *HEG1* is a regulator of heart and vessel formation [57]; and *DECR2* is involved in lipid metabolism [58], which might also be related to diabetes [59]. Overall, many of the genes annotated to the top DMPs identified in our study have biological functions related to diabetes, which suggests that DNA methylation might be involved in or be informative about the pathogenesis of diabetes.

The enrichment analysis also revealed biological pathways relevant for diabetes, such as autophagy [60], which was significantly enriched in both KEGG pathways and Reactome pathways analyses. Autophagy is a relevant regulatory signaling pathway for type 2 diabetes and is closely related to glucose and lipid metabolism, in addition to secretion of insulin. Autophagic dysfunction has been implicated in the pathogenesis of diabetes, and some diabetes therapies appear to improve autophagy in parallel with *β*-cell function, although more research is needed to clarify the specific mechanisms [61]. Additionally, TGF-*β* signaling has pleiotropic roles, including the development and function of pancreatic islet *β* cells [62], and TGF-*β*1 is known to play a role in the pathogenesis of diabetes nephropathy, a common cause of renal failure among persons with diabetes mellitus [63]. Our findings suggest that some of these processes that are known to be involved in the development and/or consequences of diabetes may be reflected in differential DNA methylation of peripheral blood immune cells.

Environmental factors such as diet and lifestyle are known to be major regulators of epigenetic marks [64]. Several studies have highlighted the association between dietary patterns and DNA methylation changes. In particular, a Mendelian Randomization study including five population-based cohorts of European, African and Hispanic participants revealed potential causal associations of diet-related CpGs with type 2 diabetes [65]. In addition, the Make Better Choices 2 study found differential patterns of DNA methylation following a healthy diet and physical activity intervention [66]. While the clinical implications of these findings are still unclear, future studies can investigate whether interventions addressing lifestyles (e.g., healthy diet, increase physical activity) modify DNA methylation changes on type 2 diabetes in populations. Beyond understanding the role of DNAm in the development of diabetes, additional work is still needed (such as longitudinal epigenetic assessments) to improve prediction of diabetes risk.

This work has some limitations. First, diabetes incidence could not be assessed with exact diagnosis dates, which is typical of prospective cohort studies that identify diabetes through examination visits. Although we know that all participants were free of diabetes diagnosis at the time of DNA methylation data collection, subclinical metabolic disorders that are related to diabetes risk were likely present in many participants, as reflected in the common signals related to baseline glucose and HOMA-IR and incident diabetes. Our study evaluated participants 45–74 years of age in a population with a high burden of diabetes, likely resulting in an evaluation late in the natural history of the disease, as reflected by the fact that increasing age was not associated with diabetes risk, which has already been described in the SHS [67]. Long prospective studies in younger populations with diabetes incidence follow-up are needed to confirm if our findings are relevant only for older populations with a high burden of diabetes or also for younger populations and populations with lower diabetes risk. In addition, future studies should assess diabetes-related DNA methylation changes over time. Of note, no DMPs were significant after multiple comparisons. However, the fact that some of the top identified genes were shared among the one marker at a time and the multiple markers at a time approaches supports the biological meaningfulness of those signals.

Strengths of this study include the high-quality DNA methylation data, measured with one of the largest microarrays available with nowadays technology, the detailed data on potential confounders and the innovative statistical methods. This is, to our knowledge, the first study that uses the ISIS-AENET tool with both continuous and dichotomous health outcomes applied to real data.

In conclusion, DNA methylation dysregulations were associated with fasting blood glucose levels and HOMA-IR, some of which were also associated with incident diabetes in the Strong Heart Study population, although no DMPs reached the significance threshold after correction for multiple comparisons. The biological functions of the genes found in the differential methylation analysis and in the bioinformatic analyses support a biological link between DNA methylation profiles, metabolic processes and diabetes risk. Further prospective and experimental studies are needed to assess the potential role of DNA methylation in diabetes.

## Supplementary Information

**Additional file 1: Fig. S1**. Flowchart of included participants in each study visit of the Strong Heart Study.

**Additional file 2: Table S1**. DMPs identified by ISIS-AENET as associated with fasting glucose. **Table S2**. DMPs identified by ISIS-AENET as associated with HOMA-IR.

## Data Availability

The data underlying this article cannot be shared publicly in an unrestricted manner due to limitations in the consent forms and in the agreements between the Strong Heart Study tribal communities and the Strong Heart Study investigators. The data can be shared to external investigators following the procedures established by the Strong Heart Study, available at https://strongheartstudy.org/. All analyses were conducted in R version 3.6.2, and all packages used are freely available in the CRAN repository.
