# Supplementary material for: Epigenetics of type 2 diabetes and diabetes-related outcomes in the Strong Heart Study
Source: Clin Epigenetics. 2022 Dec 18;14:177. doi: 10.1186/s13148-022-01392-7 (PMC9759920; doi:10.1186/s13148-022-01392-7)
Supplement: Supplementary file 1 — Additional file 1: Fig. S1. Flowchart of included participants in each study visit of the Strong Heart Study. [file 13148_2022_1392_MOESM1_ESM.docx]

**SUPPLEMENTARY MATERIAL**

**Figure 1.** Flowchart of included participants in each study visit of the Strong Heart Study.
